# Gut Microbiota and Type 1 Diabetes Mellitus: The Effect of Mediterranean Diet

**DOI:** 10.3389/fnut.2020.612773

**Published:** 2021-01-13

**Authors:** Cinzia Myriam Calabrese, Alessia Valentini, Giorgio Calabrese

**Affiliations:** ^1^PoliTO BioMed Lab, Politecnico di Torino, Torino, Italy; ^2^Dipartimento di Medicina Interna, Ospedale Madre Giuseppina Vannini, Rome, Italy; ^3^Dipartimento di Scienze e Innovazione Tecnologica, Università del Piemonte Orientale, Alessandria, Italy

**Keywords:** type 1 diabetes mellitus, gut microbiota, Mediterranean diet, short-chain fatty acids, fibers

## Abstract

Type 1 diabetes mellitus (T1DM) is a chronic autoimmune disease resulting from a complex interplay between genetic susceptibility and environmental factors. Regarding the latter, gut microbiota has a pivotal role in the pathogenesis of T1DM, by affecting intestinal permeability, molecular mimicry, and modulating innate and adaptive immune system, as described in several previous studies. The composition of the gut microbiota is largely influenced by diet. Some observational studies have shown that a low fiber intake is associated with the development of many inflammatory and immune-mediated diseases. In this context, the Mediterranean diet (MD), which is based on high consumption of cereals (preferably as whole grains), legumes, nuts, vegetables, fruits, olive oil, and fish, could play a protective role. Many of the characteristic components of MD have functional characteristics with positive effects on health and well-being. Eating habits are the main significant determinants of the microbial multiplicity of the intestine and the food components influence both microbial populations and their metabolic activities from the early stages of life. Moreover, food metabolites influence the immune response. The intestine is considered the primary site where food metabolites mediate their effects, through epithelial integrity or mucosal immunity. The compromised epithelial integrity allows the translocation of bacteria and/or the diffusion of their products, such as food antigens and lipopolysaccharides, from the intestinal lumen to the tissues, which could enhance the stimulation of immune cells, contributing to the pathogenesis of autoimmune diseases, such as T1DM. The intake of a high amount of fiber and therefore of prebiotics with MD allows the microbiota to have a good microbial balance. Moreover, as more dietary fibers are ingested, a higher amount of short-chain fatty acids (SCFAs) is produced by anaerobic gut microbiota, promoting gut homeostasis, to which also contribute tryptophan metabolites and omega-3-fatty acids. Furthermore, the higher intake of polyunsaturated fatty acids and omega-3-fatty-acids contribute to a better metabolic control. In this review we report the relationship between gut microbiota and T1DM and we explore the effects of Mediterranean diet on microbiota as a potential therapeutic strategy, aimed at preventing or delaying progression of T1DM and its complications.

## Introduction

Type 1 Diabetes Mellitus (T1DM) accounts for about 5–10% of all cases of diabetes (1) and is a chronic autoimmune disease resulting from a complex interplay between genetic susceptibility and environmental factors (2–4).

Up to 50% of genetic risk for T1DM is due to the presence of susceptibility loci within the human leukocyte antigen (HLA) regions (5–7), with a strong linkage between T1DM and genetic variations of the HLA region on the short arm of chromosome 6 (6p21) (4–6, 8, 9).

Non-HLA genes are also associated with T1DM, particularly the insulin gene region on the short arm of chromosome 11 (11p15) (4, 6) and other 58 genomic regions discovered with the genome-wide association studies (5, 6, 10). Most of these loci exert their effects acting at multiple levels in the immune system (8) and leading to an aberrant immune responsiveness (11).

Although genetic susceptibility contributes to the pathogenesis of T1DM, several non-genetic factors, such as environmental ones, are involved in the development of the disease (2).

Viral infections seem to have a pivotal role in the pathogenesis of T1DM (2, 12, 13), as reported in a systematic review and meta-analysis of the literature which showed an association between enterovirus infection and T1DM (14), and Coxsackievirus B1 was recently identified as a diabetogenic virus able to induce β-cell autoimmunity (15).

Other candidate environmental triggers include dietary factors, toxins and gut microbiota (12, 13, 16).

Many nutritional factors could contribute to the pathogenesis of T1DM (17, 18), such as cow's milk, animal protein, and fruit and berry juices (19, 20), while polyunsaturated omega-3 fatty acids have a protective role by modulating immune response (21, 22).

It has been recently supposed that gut microbiota, whose composition is largely influenced by diet (23), also plays an important role in the pathogenesis of T1DM, by affecting intestinal permeability, molecular mimicry, and modulating innate and adaptive immune system (24).

In this review we report the relationship between gut microbiota and T1DM, we explore the effects of diet, particularly of Mediterranean diet (MD), on microbiota, and we analyse its potential role as a therapeutic strategy, aimed at preventing or delaying progression of T1DM and its complications.

## Gut Microbiota

Human gut microbiota mainly consists of about 10^14^ commensals bacteria (25) belonging to seven different divisions with a high predominance of *Bacterioidetes* (particularly *Bacterioides* and *Prevotella*) and *Firmicutes*, which constitute more of 90% of the total population (26, 27), but it also includes archaea, viruses, parasites, and fungi (28). According to recent studies, gut microbiota can be separated into three robust clusters, designated as “enterotypes,” identifiable by variation in the levels of one of three main genera: *Bacteriodetes* (enterotype 1), *Prevotella* (enterotype 2), and *Ruminococcus* (enterotype 3) (27).

Gut microbiota has several roles in human health, acting as a mucosal barrier against pathogens, contributing to improve the strengthening of microvilli, providing nutrients such as the degradation of non-digestible polysaccharides and the synthesis of vitamins, maintaining metabolic homeostasis and modulating systemic immune response (25, 28–32).

Gut microbiota composition is largely influenced by diet (23). Nutrients for the gut commensals bacteria derive from the fermentation of non-digestible dietary carbohydrates (30), leading to the production of gases, smaller amounts of organic acids and short chain fatty acids (SCFAs) (26, 30, 33), such as acetate, propionate and butyrate which perform a pivotal role in human health (33). Particularly, butyrate exerts its effects on the intestinal mucosa inducing mucin synthesis and promoting tight junctions formation (34), contributing in the maintenance of physiological gut permeability. Moreover, SCFAs, the main metabolic products of fermentation, are involved in the modulation of the immune system (35, 36) by regulating the production of pro-inflammatory cytokines and chemokines (35, 37) and T-cell function (36).

## Gut Microbiota and T1DM

Several previous studies conducted in non-obese diabetic mice suggested a role of gut microbiota in the pathogenesis of T1DM (24, 38). Subsequent studies carried out on the human population also showed a strong association between gut dysbiosis and the pathogenesis in T1DM (39), suggesting that intestinal microbiota contributes to the development and progression of the disease (40). Gut dysbiosis, in fact, could contribute to the alteration of the balance between pro-inflammatory vs. tolerant signals, thus promoting the development of pancreatic autoimmunity (41).

As described by Murri et al. T1DM is associated with changes in the composition of gut microbiota (42). Particularly, it has been showed a significant difference in the amount of the two most abundant phyla of human gut microbiota, the *Bacteriodes* and the *Firmicutes* (43), with a large amount of *Bacteriodes* in T1DM (43, 44) which leads to an increase in *Bacteriodes*-to-*Firmicutes* ratio (45).

Although the alterations of gut microbiota contribute to the pathogenesis of T1DM, the exact mechanisms involved in this process are still unknown (46, 47). To date, it has been hypothesized that gut microbiota is involved in the regulation of intestinal mucosa permeability (48).

The large amount of *Bacterioides* contributes to the production of SCFAs other than butyrate (49), leading to a higher gut permeability (50) and thus contributing to the development of anti-islet cell autoantibodies (34, 51). As reported in previous studies, the increase in intestinal permeability observed in T1DM leads to microbial translocation into the circulatory system (39, 41), by inducing both a direct damage and an inflammatory immune-mediated damage of the pancreatic β-cells (52, 53).

To sustain the hypothesis of the interaction between gut dysbiosis, intestinal permeability, and host immune system, a recent study of fecal meta-proteomic showed that T1DM patients have increased intestinal inflammation, as expressed by higher levels of inflammatory proteins (galectin-3 and fibrillin-1), and altered gut permeability, as a consequence of a higher mucin degradation and lower butyrate production (54).

Nevertheless, it has been recently proposed a direct interaction between gut microbiota and pancreatic β-cells as suggested by the observation that the exocrine pancreas secretes into the intestinal tract both digestive enzymes and antimicrobial peptides (AMPs) (55), such as cathelicidins and defensins, which play a central role not only in protecting against infections but also in regulating gut microbiota (55–57) and in modulating the innate and adaptive immune system (58). Furthermore, as previously described, cathelicidin exerts trophic effect on pancreatic β-cells (57) and regulates its functions, attenuating inflammatory responses and β-cell death in diabetic subjects (59). As shown by Sun et al. gut microbiota contributes to the production of AMPs via SCFAs (60), thus gut dysbiosis may induce a lower levels of AMPc, particularly of cathelicidin and beta-defensin-1 (61), leading to the development of autoimmune diabetes (57, 60).

However, further studies are needed to better investigate the exact mechanism by which gut microbiota interacts with pancreatic β-cells and immune system.

## Mediterranean Diet

The Mediterranean Diet (MD) was first described by Prof. Ancel Keys in the 1960s (62) following the results of the epidemiological study “Seven Countries' Study,” which showed a lower incidence of cardiovascular and oncological diseases in the populations of the Mediterranean area (63, 64).

MD can be considered not only a simple diet, but also a sustainable diet, because of its low environmental impact, its attention to food and nutrition safety, and to a healthy lifestyle (65). Concerning the benefits of the MD, several studies confirmed a protective role of the MD against the development of different chronic degenerative diseases, contributing to a lower mortality risk (66, 67).

**Figure 1:**
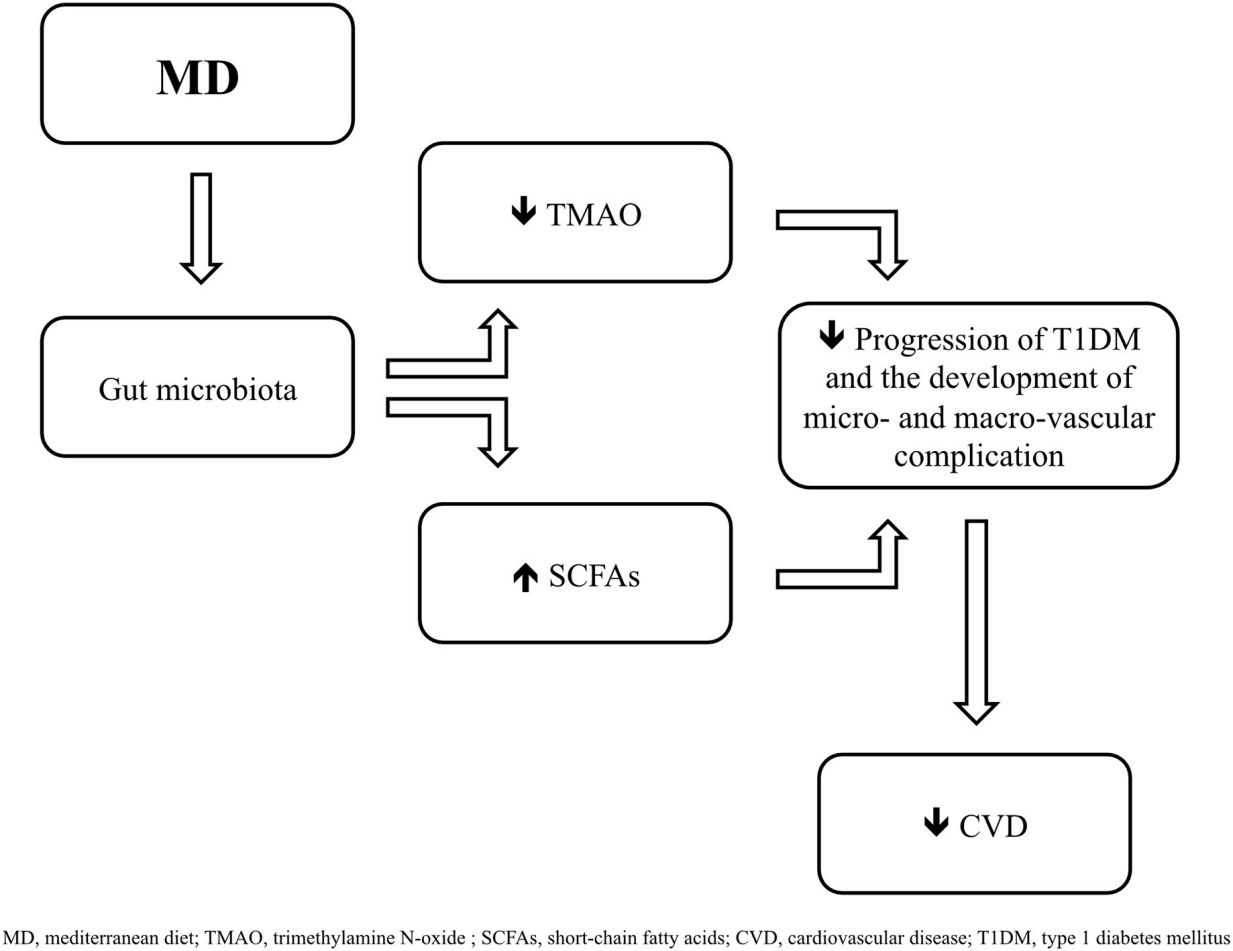
Mediterranean diet (MD) induces changes in gut microbiota by promoting the growing of taxa which contribute to a greater formation of short-chai fatty acids (SCFAs) and to a lower production of trimethylamine N-oxide (TMAO), leading to a delay in the progression of type 1 diabetes mellitus (T1DM) and in the development of cardiovascular complications.

MD is characterized by a high intake of cereals, legumes, vegetables, fruit, nuts, fish and an elevated consumption of olive oil, a moderate alcohol intake, and a low consumption of red meats and saturated fats (68). As reported in previous studies, in fact, the MD consists of a diet rich carbohydrates (55–60% of total energy, of which <15% are sugars), proteins (15% of total energy, with a prevalence of vegetables origin) and fibers (25 g/day), and low in lipids (30% of total energy, of which about 19% derive from monounsaturated fat, 5% from polyunsaturated fat, and <7% from saturated fat) (69–71). Relative to lipids, the main source of dietary fat in MD is represented by extra virgin olive oil (EVOO) (71, 72), whose consumption is associated with a reduction in the development of cardiovascular diseases (72–74) and, as recently shown, with the modulation of gut microbiota (74–76), contributing to a relative abundance of *Firmicutes* compared with *Bacterioidetes* (77). EVOO is predominantly composed by monounsaturated fatty acids (MUFAs), polyunsaturated fatty acids (PUFAs), such as linoleic and α-linolenic acids, and saturated fatty acids (SFA), mainly palmitic and stearic acids, and it also contains vitamin E and polyphenols, such as hydroxytyrosol (78). The beneficial effects of EVOO can also be associated with the presence of 3,3-dimethyl-1-butanol (DMB), a structural analog of choline, involved in the inhibition of trimethylamine N-oxide (TMAO) formation (79), a metabolite of phosphatidylcholine and L-carnitine, which production is mediated by gut microbiota (80) (Figure 1). As reported in several studies, higher levels of TMAO contribute to an increased cardiovascular risk (81, 82), and in type 1 diabetic subjects are associated with mortality, coronary events, stroke, heart failure and poor renal function (83).

MD is based on a large daily consumption of fiber (71), a very present element among plant foods, with different chemical structures, and resistant to human enzymatic degradation (84). Dietary fiber is distinguished in two main classes: polysaccharides, including cellulose and non-cellulose substances, the latter mainly represented by hemicellulose and pectin, and lignin (84). Moreover, fiber can be divided into soluble fiber, such as pectin, and insoluble ones (85).

Dietary fiber resists the action of the upper gastrointestinal human enzymes and reaches the large intestine where the local microbiota induces its fermentation, leading to the production of SCAFs (84, 86, 87) that, as described above, contribute to the energy metabolism of the host and modulate its immune system (88).

Some dietary fiber can be classified as prebiotics (89). Gibson G. R. and colleagues defined a prebiotic as “a non-digestible food ingredient that beneficially affects the host by selectively stimulating the growth and/or activity of one or a limited number of bacteria in the colon, and thus improves host health” (90). Subsequently, as shown by the same researcher (91), prebiotics has been defined as “a selectively fermented ingredient that allows specific changes, both in the composition and/or activity in the gastrointestinal microflora, that confer benefits” (86).

Prebiotics are composed of carbohydrates, mainly oligosaccharides, which resist to the digestion in the upper gastrointestinal tract and are fermented in the colon, producing SCFAs and favoring the growth of beneficial bacteria, particularly *Bifidobacterium, Faecalibacterium prausnitzii, A. Muciniphila*, and *Ruminoccoccus bromii* (28). The main target of prebiotics, in fact, are some intestinal commensal bacteria, such as *Lactobacilli* and *Bifidobacteria*, also known as probiotics (86, 92).

Finally, probiotics have been defined as “live microorganisms that confer a health benefit to the host, when administered in adequate doses” (93). In fact, probiotics can prevent intestinal infections by inhibiting the adhesion of pathogens to the intestinal mucosa, are involved in the maintenance of the physiological gut permeability through the increased production of mucin and the strengthen of tight junctions, and modulate innate and adaptive immune system (92, 94).

The beneficial effects of MD could also be related with its ability to impact the composition of the gut microbiota, leading to the production of several metabolites able to promote metabolic and molecular health (95–97). In this regard, it has been described that MD is associated with a lower *E. coli* fecal counts and a higher culture-based *Bifidobacteria:E. coli* ratio, increased levels of *C. albicans*, greater amount of SCFAs (98), and with a higher levels of *Faecalibacterium prausnitzii* and *Clostridium cluster XVIa* (99). According to Tindall et al. (100), further researches are needed to investigate the relationship between MD and gut enterotypes, although a study carried out by Wu et al. showed that *Bacteroides* “enterotype” was highly associated with animal protein and saturated fat consumption, while *Prevotella* “enterotype” was linked to high values of carbohydrates and simple sugars (101).

As described above, MD is characterized by a higher intake of plant foods (71), which contain microconstituents with significant biological activity, known as bioactive compounds or phytochemicals (102). According to Kitty et al. bioactive compounds are “extra-nutritional” constituents naturally present in small quantities in the food matrix (103) and include flavonoids, resveratrol, phytoestrogens, lycopene, phytosterols, dietary fibers, isothiocyanates, and monoterpenes (104). Most of these compounds have antioxidant activity (102), contributing in the prevention of chronic disease (102, 105).

## Mediterranean Diet and T1DM

T1DM is associated with high cardiovascular risk (106), which is largely influenced by environmental factors, such as diet (107).

It is known that MD, rich in monounsaturated fat and fiber, is protective against cardiovascular risk (108). This observation led to the recommendation of a higher consumption of saturated fatty acids (SFA) and dietary fiber (109), as also suggested by several studies demonstrating an inverse association between dietary fiber and cardiovascular risk (110, 111). Moreover, a multicentric study carried out in a population of European type 1 diabetic patients examined the effects of SFA and dietary fiber intake on the onset of cardiovascular disease, and showed that an increased consumption of fiber, especially soluble fiber, can prevent both cardiovascular and all-cause mortality risk in these patients (112). Finally, a recent study confirmed that MD reduces cardiovascular risk but also proved that it improves glycemic control in type 1 diabetic patients (113).

## Gut Microbiota, Mediterranean Diet, and T1DM

It has been proved that diet largely influences human gut microbiota (114, 115). MD, which recommends a grate intake of whole grains, fruit, vegetables, legumes, fiber and unsaturated fats, contributes to a favorable microbiota, and gut-related metabolomic profile (97, 116). In this regard, a study carried out by De Filippis et al. showed that a high-level adherence to the MD was associated with an increased amount of *Prevotellaceae* and a reduced *Firmicutes*-to-*Bacteroidetes* ratio, and with a greater levels of SCFAs and a lower concentrations of TMAO (97). Accordingly, a recent study carried out in an elderly population, showed that MD alters gut microbiota, promoting the growing of taxa such as *Faecalibacterium prausnitzii, Bacteroides thetaiotaomicron*, and *Prevotella copri*, which contribute to the formation of SCFAs (117).

Furthermore, MD-induced changes in gut microbiota promote a greater production of SCFAs, and particularly of butyrate, which exert an immunoregulatory effects (118), as described above, and regulate oxidative stress and inflammation in the gut and in others peripheral tissues (119, 120).

Gut microbiota is also modulated by probiotics, which contribute to maintenance of physiological intestinal permeability, thus interfering with autoimmune pancreatic β-cell destruction (121), as shown in a study carried out in a mice model of T1DM, in which the administration of *Lactobacillus johnsonii* delayed the development and the progression of the disease (122).

## Conclusions

The interaction between diet, intestinal microbiota and the development and progression of T1DM could lead to the hypothesis that MD can modify both the onset and the progression of the disease by modulating the intestinal microflora and reducing the associated pro-inflammatory profile.

The high intake of fibers could especially contribute to a favorable ecology of the microbiota which increases the production of SCAFs, mainly of butyrate, leading to the maintenance of physiological intestinal permeability and thus regulating the response of the immune system. In addition, the large amount of fiber improves glycemic control and fat absorption, slowing the progression of the disease and delaying the development of cardiovascular complications.

Nevertheless, further researches are needed to evaluate the effect of MD on the gut microbiota of type 1 diabetic patients and to investigate the potential beneficial effects of a greater adherence to the MD in this population, to better comprehend its eventual prophylactic and therapeutic role. Moreover, it could be interesting to evaluate the effects of a higher intake of probiotics in type 1 diabetic subjects and in their microbiota, to better understand the role of probiotics, as therapeutic agents, in the modification of the composition of the human gut microbiota and in the onset and progression of diabetic disease.

To date, according with the data currently published, we recommend a greater adherence to MD, favoring a large consumption of dietary fiber and probiotics and we suggest more researches in this field, also to find new probiotic's formulations, with the aim of ameliorate the adhesion to the MD and to take healthy advantages of its consumption.

## Author Contributions

CC, AV, and GC designed the review, performed the literature analysis, and wrote the manuscript. GC critically revised the text and gave a substantial scientific contribution. All authors approved the final version of the manuscript for publication.

## Conflict of Interest

The authors declare that the research was conducted in the absence of any commercial or financial relationships that could be construed as a potential conflict of interest.
